# Evolutionary medicine and psychiatry in medical education: a scoping review

**DOI:** 10.1093/emph/eoag019

**Published:** 2026-10-05

**Authors:** Gurjot Brar, Eimear Ní Mhuircheartaigh, Krystal Poobalan, John McFarland, Henry O’Connell

**Affiliations:** School of Medicine, University of Limerick, Limerick, Ireland; Department of Psychiatry, Department of Psychiatry, Trinity College Dublin, Dublin, Ireland; School of Medicine, University of Limerick, Limerick, Ireland; School of Medicine, University of Limerick, Limerick, Ireland; Department of Psychiatry, Midlands Regional Hospital, Portlaoise, Co Laois, Ireland; School of Medicine, University of Limerick, Limerick, Ireland; School of Medicine, University of Limerick, Limerick, Ireland; Department of Psychiatry, Midlands Regional Hospital, Portlaoise, Co Laois, Ireland

**Keywords:** psychiatry, medicine, evolution, education

## Abstract

**Background:**

Evolutionary medicine (EvMed) and evolutionary psychiatry (EvPsych) apply principles of evolutionary biology to deepen understanding of disease vulnerability, mental health, and clinical reasoning. Despite growing conceptual maturity and relevance across biomedical domains, the extent and nature of their integration into medical and psychiatric education remain unclear.

**Objective:**

This scoping review aims to map the education-focused literature on EvMed and EvPsych across undergraduate, postgraduate, and continuing professional training contexts, and to characterize the types of educational initiatives, frameworks, and evaluation strategies described.

**Methods:**

A scoping review methodology was employed to search multiple databases and supplementary sources systematically. Eligible sources included conceptual curriculum frameworks, descriptive studies of curricular provision or learner attitudes, and interventional or evaluative educational studies. Data were charted and categorized by educational context, study design, and primary educational contribution. Findings were synthesized narratively.

**Results:**

The final 19 articles include conceptual advocacy, curriculum mapping studies, and a smaller number of implementation and evaluation reports. While there is broad agreement regarding the theoretical value of evolutionary perspectives for enhancing clinical understanding and integrative reasoning, formal curricular integration remains uneven. Reported barriers include limited curricular time, faculty expertise, and perceived controversy. Existing educational interventions are typically modular, case-based, and of short duration, with outcomes primarily focused on learner knowledge and attitudes.

**Conclusions:**

EvMed and EvPsych possess a coherent conceptual foundation but remain variably embedded within formal training pathways. Future work should prioritize rigorous evaluation, competency alignment, and scalable implementation strategies to support sustained curricular integration.

**Lay summary:**

Evolutionary medicine (EvMed) applies evolutionary biology to explain why humans are vulnerable to disease. This review maps how EvMed and evolutionary psychiatry are taught in medical training. Although widely valued, integration remains uneven. Stronger evaluation and curriculum development are needed to embed evolutionary thinking in clinical education.

## INTRODUCTION

Evolutionary medicine (EvMed) applies evolutionary biology to explain human vulnerability to disease, complementing proximate mechanisms with ultimate explanations grounded in natural selection, trade-offs, constraints, mismatch, and host–pathogen coevolution [1–3]. Evolutionary psychiatry (EvPsych) extends this framework to mental health, using evolutionary theory to examine the origins and persistence of emotions, behaviour, and psychological vulnerabilities [4–6]. Together, these approaches offer an integrative account of health across physical and mental domains [2, 4].

Evolutionary perspectives have increasingly been linked to major challenges in medicine, psychiatry, and public health, including antimicrobial resistance, cancer as somatic selection, inflammatory dysregulation, life history processes, and environmental mismatch [2, 3]. Tinbergen’s ‘four questions’ (mechanism, ontogeny, function, phylogeny) provide a widely cited scaffold for integrating proximate and evolutionary explanation in behavioural science [5, 7]. The field now positions evolutionary thinking as a unifying framework capable of integrating fragmented biomedical knowledge [2, 4, 8], with calls for its inclusion in medical curricula worldwide [1, 9]. EvPsych receives particular emphasis here reflecting the authors' backgrounds in psychiatry and a recognition that the EvPsych education literature is not yet sufficiently developed to support a standalone scoping review. Much of the relevant evidence base derives from the broader EvMed literature, the principles and pedagogical approaches of which are largely transferable to psychiatric training contexts. The results therefore draw on both bodies of literature, encompassing multiple medical subdisciplines alongside psychiatry-specific contributions.

However, the education-focused literature in EvMed and EvPsych spans multiple diverse forms. Curriculum proposals, competency arguments, teaching resources, learner-facing conceptual frameworks, and empirically evaluated educational initiatives are distributed across undergraduate, postgraduate, and continuing professional education contexts. Such diversity creates a familiar synthesis problem in health professions education which is that the evidence base may be substantial yet heterogeneous in aims, designs, and outcomes, proving difficult to determine what has been attempted, where it has been implemented, and what has been measured. This challenge is amplified by the methodological variability typical of education research, where outcomes range from knowledge and attitudes to performance-based assessments and downstream behavioural indicators, and where traditional quality appraisal tools may be difficult to apply uniformly.

Scoping review methodology is therefore appropriate to map the extent, nature, and characteristics of this diverse literature [10]. Following PRISMA Extension for Scoping Reviews (PRISMA-ScR) guidance [11], this review aims to: (a) characterize the types of educational publications and activities reported; (b) describe how and where evolutionary perspectives are represented across training contexts; and (c) summarize the educational approaches and outcomes described or evaluated.

## METHODS

### Study design

A scoping review methodology was employed to map the nature, extent, and characteristics of the literature on EvMed and EvPsych in medical education. This approach was chosen because the field contains substantial heterogeneity in study designs, outcomes, and objectives [10]. The review was conducted and reported in accordance with the PRISMA-ScR. Consistent with PRISMA-ScR guidance, no formal critical appraisal of included sources was undertaken. Given the inclusion of conceptual frameworks, descriptive studies, and heterogeneous educational interventions, application of a single quality appraisal instrument such as MERSQI was not felt appropriate [12]. While EvMed encompasses applications across many areas of healthcare, EvPsych was given particular attention in the present review because of the authors' disciplinary interest and because psychiatry presents unique conceptual challenges regarding the integration of biological, psychological, and social explanations of illness. EvPsych explicitly addresses questions of ultimate causation and vulnerability to mental disorders, complementing proximate biological and psychosocial accounts. However, the scope of this review extends beyond psychiatry and includes educational initiatives from across EvMed more broadly.

### Formulation of research questions

The formulation of the questions was guided by the Population–Concept–Context framework recommended for scoping reviews. The population of interest comprised medical students, postgraduate medical and psychiatric trainees, clinicians, and medical educators; the concept was EvMed and EvPsych education; and the context was undergraduate medical education, postgraduate training, and continuing professional education.

Based on this framework, the review addressed the following research questions:

1. **What types of education-focused literature exist on EvMed and EvPsych in medical and psychiatric training?**
2. **How is EvMed and EvPsych currently represented within medical and psychiatric education?**
3. **What educational interventions, frameworks, or pedagogical approaches have been described or evaluated, and what outcomes have been reported?**

### Eligibility criteria

#### Inclusion criteria

Sources were included if they involved medical students, postgraduate trainees, clinicians, or medical educators, and explicitly addressed EvMed and/or EvPsych within medical or psychiatric education. Eligible contexts included undergraduate, postgraduate, and continuing professional education. Studies had to have an explicit educational focus, such as describing or evaluating an educational intervention, reporting educational research (e.g. surveys, curriculum mapping, needs assessments), or providing structured curriculum guidance (e.g. syllabi, learning objectives, or frameworks). Publications were limited to English-language sources published from 1990 onwards.

#### Exclusion criteria

Sources were excluded if they addressed EvMed or EvPsych without a clear educational focus, or were purely clinical, theoretical, or narrative without explicit curricular relevance. Studies focused on non-medical education (e.g. undergraduate biology, public health, or psychology without a medical training focus) were also excluded. Editorials, opinion pieces, and reviews lacking structured educational outputs were omitted. Conceptual papers were included only if they contained explicit curriculum-related content; otherwise, they were treated as background literature.

### Information sources

The following databases were systematically searched: PubMed/MEDLINE, Scopus, PsycINFO, ERIC. To ensure comprehensive coverage, supplementary searching was undertaken using: Google Scholar, MedEdPORTAL, and reference lists following screening of included studies. Google Scholar was searched using the same core search terms as the database searches, with results screened to the first 100 records, consistent with established scoping review practice for grey literature. MedEdPORTAL was searched using the terms “evolutionary medicine” and “evolutionary biology” with an education filter applied. Reference lists of all included studies were hand-searched to identify any additional sources not captured through database searching. Grey literature sources were subject to the same inclusion and exclusion criteria as peer-reviewed sources, requiring an explicit educational focus and relevance to medical or psychiatric training. Systematic searching beyond these specified supplementary sources was not undertaken, and this constitutes a limitation of the review.

### Search strategy

Search strategies were developed iteratively and adapted for each database using a combination of controlled vocabulary and free-text terms. Core search concepts included: *Evolutionary medicine, evolutionary psychiatry, evolutionary biology, medical education, psychiatric education, training, curriculum, teaching, medical students, psychiatrists, trainees, residents*. A detailed example of database-specific search strings is provided in the Supplementary Material S1.

### Selection of sources of evidence

All records were exported to Zotero for reference management, and duplicates were identified and removed prior to screening. Screening was conducted in two stages by two researchers (G.B. and H.O.’C.). In the first stage, titles and abstracts were screened to assess relevance. In the second stage, three researchers (G.B., E.M., K.P.) reviewed full texts against the eligibility criteria, with particular attention to whether the source included an evaluated educational intervention, reported descriptive or observational educational data, or provided structured curriculum guidance. Sources that did not meet the education-focused inclusion criteria were classified as background literature.

### Data charting process

For each included study, the following data were extracted:

- Author(s) and year
- Country or setting
- Learner group or participants
- Educational context (undergraduate, postgraduate, continuing education)

### Study design

- Educational focus (EvMed, EvPsych, or both)
- Key educational outcomes or contributions
- Primary evidence type.

**Classification of evidence types**

- **Type 1: Conceptual/theoretical/position papers**
  Papers proposing frameworks, syllabi, learning objectives, consensus principles, or arguments for curricular inclusion
- **Type 2: Descriptive/observational educational research**
  Studies using surveys or curriculum mapping
- **Type 3: Interventional/evaluative educational studies**
  Studies evaluating implemented educational interventions

### Synthesis of results

We used a mixed-methods approach combining descriptive statistics and thematic analysis to synthesize data on the integration of evolutionary perspectives in medical education [13]. Descriptive statistics summarized publication type, study design, and geographical distribution. Thematic analysis identified recurrent concepts, pedagogical approaches, and reported challenges in incorporating EvMed/EvPsych into curricula. To enhance analytic rigour, multiple authors independently conducted the analysis and reached consensus through discussion, reducing individual bias. Two subject-matter experts in EvMed and psychiatry education were consulted during selection and synthesis to support interpretation and strengthen the credibility of the findings [13].

### Ethics

Ethical approval was not required as this review is based on published literature.

## RESULTS

### Basic overview

The search strategy yielded 68 records, comprising 58 records identified through database searching and 10 records identified through supplementary sources. After removal of duplicates, 54 unique records remained for screening. Title and abstract screening resulted in the exclusion of four records. Fifty full-text articles were assessed for eligibility, of which 31 were excluded because they did not meet education-focused inclusion criteria or were purely theoretical/conceptual without structured educational outputs. Nineteen sources of evidence met the inclusion criteria and were included in the final scoping review (Fig. 1).

**Figure 1:**
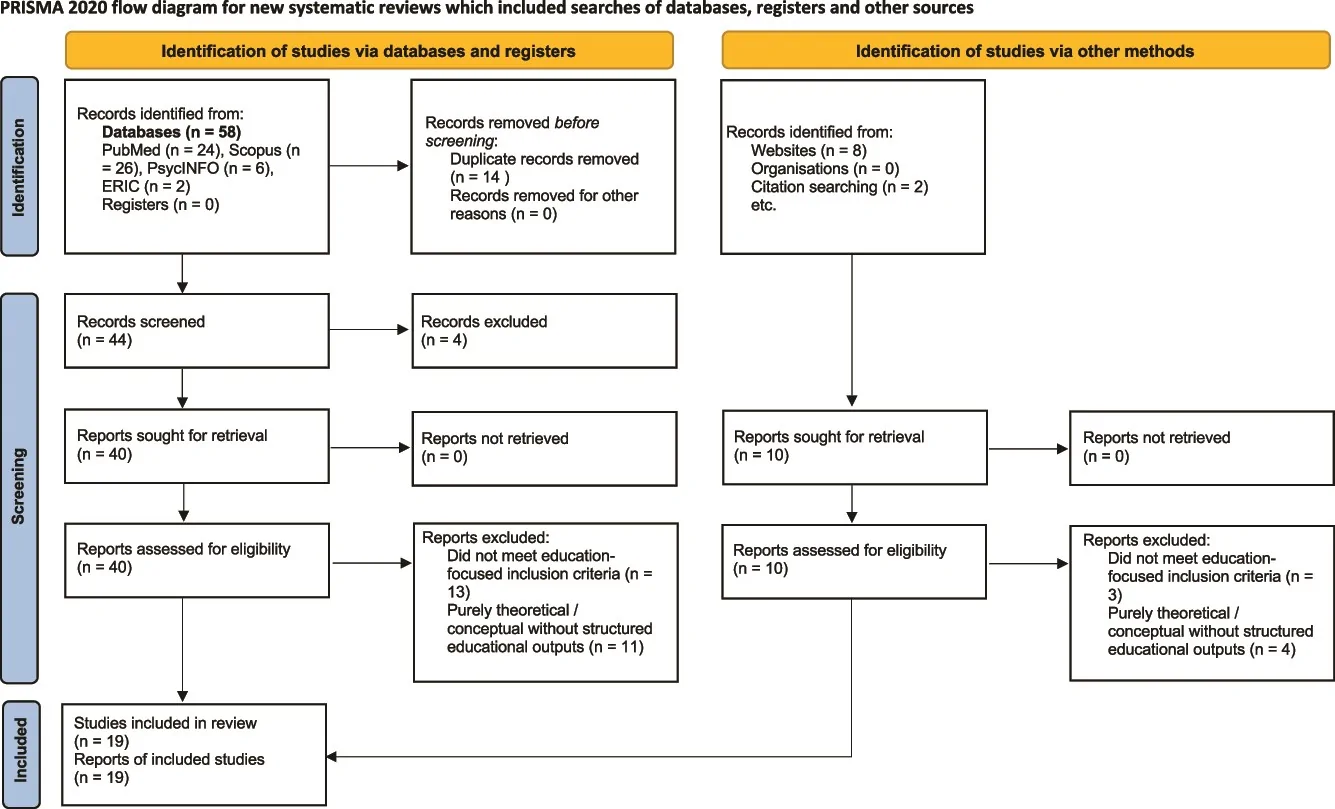
PRISMA-ScR flow diagram showing the identification, screening, eligibility assessment, and inclusion of sources in the scoping review.

### Characteristics of included studies

Across the dataset, the literature spanned 1995–2023, with a clear concentration of publications in the last 10–15 years. The included studies were geographically diverse, though most commonly represented the UK and North America, alongside examples from Venezuela, Pakistan, Norway, Germany, and Australia/Switzerland.

### Classification of evidence types

Across the 19 included papers, three broad types of contributions were identified: [1] conceptual and consensus frameworks that specify *why* EvMed/EvPsych matters and *what* should be taught; [2] status/attitudes and curriculum mapping studies that describe the *extent of current teaching* and perceived barriers; and [3] implementation and evaluation papers that describe and/or evaluate specific teaching interventions and assessment tools (see Tables 1–3).

**Table 1:** Type 1: Conceptual, theoretical and position papers proposing frameworks, syllabi, learning objectives, consensus principles, or arguments for curricular inclusion (*n* = 8).

Author(s), Year	Country/setting	Educational context (UG/PG/CPD)	Learners/participants	Study design	Sample size	Educational focus	Evidence type	Key educational contribution
Baptista, 1995	Venezuela	PG Psychiatry	Psychiatry residents	Course description	N/A	Evolutionary psychiatry teaching	Type 1	Description of an EvPsych teaching course
Charlton, 1997	UK	UG	Medical educators	Syllabus proposal	N/A	EvMed curriculum structure	Type 1	Proposes EvMed Syllabus
Bolnick *et al*., 2017	USA/International	UG	Medical educators	Learning objectives framework	N/A	Integrating evolution into medicine	Type 1	Proposes EvMed LOs
Grunspan *et al*., 2018	International	UG	EvMed experts	Delphi consensus	N/A	Core principles of EvMed	Type 1	Delphi study arriving at core principles of EvMed
Graves *et al*., 2016	USA	UG	Medical trainees	Learning objectives framework	N/A	Integrating evolution into medicine	Type 1	Maps concepts, LOs, and competencies in EvMed
Nesse *et al*., 2010	International	UG	Medical students	Position paper/curriculum framework	N/A	Evolution as basic science	Type 1	Proposes curricular principles, learning objectives, and educational sequencing
Abed *et al*., 2019	UK/International	PG	Psychiatry Residents	Conceptual framework	N/A	Evolution in psychiatry training	Type 1	Proposes LOs for EvPsych based on core principles
Mysterud, 1998	Norway	UG	UG	Description of curriculum inclusion	N/A	Integrating evolution into medicine	Type 1	Description of curriculum inclusions across Norway

Abbreviations: UG, undergraduate; PG, postgraduate; CPD, continuing professional development; EvPsych, evolutionary psychiatry; EvMed, evolutionary medicine; LOs, learning objectives; N/A, not available.

**Table 2:** Type 2: Descriptive and observational educational research using surveys or curriculum mapping (*n* = 6).

Author(s), year	Country/setting	Educational context (UG/PG/CPD)	Learners/participants	Study design	Sample size	Educational focus	Evidence type	Key educational contribution
Hidaka *et al*., 2015	USA	UG	Medical school deans	National survey	60 deans	Status of EvMed in curricula	Type 2	Surveyed curricular coverage of evolutionary medicine
Grunspan *et al*., 2019	USA	UG	Medical schools	Curriculum mapping	101 EvMed instructors	EvMed course prevalence	Type 2	Mapped prevalence of evolutionary medicine teaching
Power *et al*., 2020	USA	PG/CPD	Ob/Gyn clinicians	Cross-sectional survey	229 OBGYNs	Evolution in women’s health training	Type 2	Assessed attitudes toward evolution in women’s health training
Yousuf *et al*., 2011	Pakistan	UG	Medical students	Survey	299 students	Acceptance of evolution/EvMed	Type 2	Assessed attitudes toward evolution in medical students in Pakistan
Downie, 2004	UK	UG	Medical educators/students	Educational survey	UK medical schools (*n* = 16); first-year medical students (*n* = 223); third-year medical students completing an optional evolutionary medicine (*n* = 72)	Evolution in medical curricula	Type 2 (Type 3)	A survey of the coverage of evolutionary biology in UK undergraduate medical curricula. A survey of medical students' attitudes to evolution. A special study module on Evolution in Health and Disease and student—institutional survey + students + intervention subgroup responses to it.
Nesse & Schiffman, 2003	USA	UG	Medical school deans	National survey		Evolution in medical curricula	Type 2	Surveyed curricular coverage of evolutionary medicine

Abbreviations: UG, undergraduate; PG, postgraduate; CPD, continuing professional development; EvPsych, evolutionary psychiatry; EvMed, evolutionary medicine; OBGYN, obstetrics and gynaecology.

**Table 3:** Type 3: Interventional and evaluative educational studies evaluating implemented educational interventions (*n* = 5).

Author(s), year	Country/setting	Educational context (UG/PG/CPD)	Learners/participants	Study design	Sample size	Educational focus	Evidence type	Key educational contribution
Baptista *et al*., 2012	Venezuela	UG, PG, and CPD	Physicians, residents, med students	Pre–post pilot study	109	Evolution seminar	Type 3	Evaluation of an EvMed seminar
Abbott and Abboud, 2006	UK	UG	Medical students	Special study module + evaluation	18	Evolution in health and disease	Type 3	Evaluation of a 4-week elective course in evolutionary medicine for fourth-year students
Rühli *et al*., 2016	Switzerland and Australia	UG and PG	Medical students (and other students)	Course description and evaluation, module implementation	Approx 100	Evolutionary medicine modules	Type 3 (Type 1)	Three courses of the EvMed taught at the University of Zurich and the University of Adelaide are presented by giving their general descriptions, full curricula, and the results of anonymized student evaluations.
Furtwängler *et al*., 2023	Germany	Mixed UG and PG	Medical students and lecturers	Tool evaluation	101 student survey respondents (67 in 2021; 34 in 2022) and 15 lecturers. 424 students across five courses (including one medical faculty course).	‘Mummy Explorer’ digital tool (EvMed)	Type 3	Educational tool evaluation using post-exposure questionnaires
Misheva *et al*., 2023	North America	UG	Medical students	Ax Tool evaluation	499 students	EvMed	Type 3	Creation and validation of an assessment tool for EvMed

Abbreviations: UG, undergraduate; PG, postgraduate; CPD, continuing professional development; EvPsych, evolutionary psychiatry; EvMed, evolutionary medicine.

#### Type 1: Conceptual, theoretical, and position papers (*n* = 8)

These papers primarily aimed to legitimize EvMed as a foundational explanatory science for medicine and propose curricular structures, core principles, and educational rationales. They included an early syllabus proposal [14], learning objectives for medical education [15], a Delphi-derived set of core principles [16], and pedagogical arguments emphasizing evolutionary thinking as a method for higher-order reasoning [17] and as a basic science [1]. Psychiatry-focused conceptual work argued for evolutionary biology as essential to psychiatric training [9, 18]. One national/contextual review traced the historical emergence and curricular presence of Darwinian medicine in Norway [19].

#### Type 2: Descriptive and observational educational research (*n* = 6)

These papers documented limited and uneven incorporation of evolutionary biology/EvMed within medical education, using surveys or systematic mapping. Two studies addressed North American medical school curricula across time [20, 21]. A similar study focused on the UK [22]. One study mapped undergraduate course availability in the United States [23]. Two studies examined acceptance/attitudes in specific learner or practitioner groups: Pakistani medical students [24] and practising obstetrician–gynecologists in the United States [25].

#### Type 3: Implementation, evaluation, and measurement (*n* = 5)

These papers described or evaluated EvMed teaching in practice: a brief pre–post seminar across multiple clinician groups and medical students [26], an introductory medical-school intervention described from a curricular implementation perspective [27], evaluated case-based modules implemented in Australia and Switzerland [28], and two studies that sought to develop an infrastructure for scalable teaching; an open-access digital teaching tool [29], and a validated assessment instrument (EvMed assessment tool) [30].

### Narrative thematic synthesis

#### Theme 1: EvMed is repeatedly positioned as an ‘integrative explanatory science’ rather than additional content

A dominant thread across the conceptual literature was that evolutionary thinking provides *ultimate* explanations (why vulnerability exists) that complement proximate biomedical mechanisms (how disease occurs). Charlton [14] proposed one of the earliest structured syllabi explicitly organizing medical topics through evolutionary concepts such as trade-offs, constraints, mismatch, and host–pathogen coevolution.

Subsequent frameworks reinforced this integrative claim, but with increasing emphasis on curriculum design and *assessability*. Nesse *et al*. [1] framed evolutionary biology as a basic medical science with learning goals mapped to clinical domains. Bolnick *et al*. [15] translated this rationale into consensus-oriented learning objectives aligned to core evolutionary principles and clinically relevant competencies, explicitly arguing that clear objectives are required to legitimize EvMed within crowded curricula and to enable assessment. Consistent with this, Grunspan *et al*. [16] used Delphi methods to arrive at 14 core EvMed principles.

Relatedly Graves and colleagues argued that EvMed should be taught not merely as knowledge, but as a *reasoning method* that promotes higher-order thinking and synthesis. Graves *et al*. [17] explicitly linked evolutionary reasoning to educational theory (e.g. Bloom’s taxonomy) and argued that evolutionary explanation supports transfer and clinical reasoning across levels from genes to populations.

#### Theme 2: Lack of widespread adoption despite recognized value

Status and survey studies consistently reported that curricular inclusion lags behind perceived importance, though this pattern was documented most explicitly in North America. In the earlier North American dean survey, Nesse and Schiffman [21] reported that while many deans recognized evolutionary biology as important, formal teaching remained minimal and rarely required. More than a decade later, Hidaka *et al*. [20] reported increased coverage compared with earlier estimates but still a notable gap between perceived importance and actual teaching (reported as an average gap of ~21%), with ‘lack of faculty expertise’ and ‘limited curricular time’ as prominent barriers. Furthermore, nearly half of responding schools anticipated potential controversy if evolutionary content was expanded. In the UK, Downie [22] provided more detailed mixed evidence: a national survey suggested that only 37% of UK medical schools included any teaching on evolution (often optional), and a student survey indicated that approximately 10% rejected long-term evolution. This rejection being associated with religious beliefs and linked to attitudes toward clinically relevant issues such as antibiotic resistance and intergenerational responsibility.

Evidence from outside the UK/USA further underscored that ‘acceptance and context’ can be material constraints. Yousuf *et al*. [24] found that Pakistani medical students had limited understanding and a lower acceptance, particularly regarding human evolution associated with religious and cultural factors shaping acceptance despite perceived relevance to medicine (e.g. antibiotic resistance).

One national historical review [19] described Norwegian EvMed teaching as emerging through optional courses and individual academic ‘champions’ rather than formal mandates, and characterized its curricular presence as ‘marginal and fragile’, again attributing limited integration to crowded curricula, limited expertise, and perceptions that evolutionary explanations are abstract or insufficiently clinical.

#### Theme 3: Where EvMed is taught, it is often situated outside core medical curricula

The most detailed mapping of undergraduate course availability was provided by Grunspan *et al*. [23], who reported that dedicated EvMed instruction in the United States was concentrated in research-intensive (R1) universities, with courses commonly housed in biology or anthropology departments and taught by non-clinicians. Downie [22] similarly described UK medical school coverage as frequently optional, and proposed a dual strategy: brief core exposure supplemented by optional in-depth modules (with involvement from evolutionary biologists). This ‘core + elective’ pattern is also consistent with how Mysterud [19] characterized Norwegian uptake Power *et al*. [25] provided data among 229 practising obstetrician–gynecologists surveyed, most considered evolutionary biology important/vital for medical students (64.4%), and a substantial majority supported exposure during residency (81.1%; optional or required). At the same time, respondents recognized crowding and tended to prefer integration into existing teaching rather than new standalone requirements.

#### Theme 4: Implemented interventions emphasize clinical linkage, impose modest time demands, and use interactive/case-based formats

Among Type 3 studies, evaluated teaching interventions shared a recurring implementation logic. They all aimed to keep teaching time feasible, anchor concepts in concrete clinical problems, and use interactive or case-based learning.

Baptista *et al*. [26] evaluated a two-hour interactive seminar delivered to multiple clinician groups and sixth-year medical students without prior formal training in evolutionary theory. They reported overall knowledge gains and high perceived relevance, but also noted that psychiatrists showed smaller improvements and lower perceived relevance than other specialists. Although it is just one study, this highlights potential specialty-level differences in engagement that may warrant tailored approaches.

Rühli *et al*. [28] reported the feasibility of embedding short interactive modules within existing curricula in Australia and Switzerland and described improvements in student understanding and perceived relevance, with learners reporting enhanced conceptual coherence and clinical reasoning rather than being laboured with ‘extra’ content.

Abbott and Abboud [27] similarly argued that student engagement and perceived relevance increase when evolutionary principles are explicitly linked to clinical examples and recommended early introduction and curricular embedding rather than isolation.

Digital delivery emerged as a more recent implementation strategy aimed at scalability. Furtwängler *et al*. [29] evaluated an open-access self-regulated online tool (‘The Mummy Explorer’) for medical and health-science students, reporting high engagement/usability in connecting evolutionary explanations to disease patterns. The authors emphasized flexibility for integration into crowded curricula.

#### Theme 5: Movement toward standardization—Defining ‘what to teach’ and ‘how to measure it’

Across the dataset, a clear trajectory was visible from early syllabus arguments toward consensus-based specification of content and then toward measurement.

Grunspan *et al*. [16] addressed the lack of agreement on essential content by arriving at 14 core principles by Delphi consensus. Bolnick *et al*. [15] also operationalized the field by providing learning objectives mapped to competencies, explicitly linking objectives to legitimacy and assessment within constrained curricula. Misheva *et al*. [30] then addressed the measurement gap directly by developing and validating an EvMed assessment aligned to established core principles, positioning it as a pre/post evaluation tool and as an assessment resource that can support curriculum improvement and comparison.

#### Theme 6: Psychiatry and women’s health are presented as particularly ‘high-yield’ domains for evolutionary framing

Two included conceptual papers [9, 18] argued that psychiatry is especially suited to evolutionary approaches because of its focus on behaviour, emotion, and social functioning, and because ultimate explanations can restore conceptual coherence where proximate neurobiological accounts alone may fragment understanding.

However, the only evaluated teaching intervention in the summaries that directly compared psychiatrists with other clinician groups [26] suggested psychiatrists may demonstrate comparatively smaller short-term gains and lower perceived relevance following brief generic instruction, implying that specialty-tailored delivery may be important in psychiatric contexts.

In contrast, Power *et al*. [25] focused on obstetrics and gynecology and presented specialty-specific rationale (strong selective pressures and reproductive trade-offs) alongside attitudinal data indicating broad support for evolutionary exposure during residency and perceived importance for medical students. They also recognized feasibility constraints and preferred integration within existing structures.

#### Summary of results

Taken together, the included literature suggests a field characterized by (a) strong conceptual agreement that evolutionary thinking can unify medical understanding and support higher-order reasoning, (b) repeated documentation that curricular integration remains limited and uneven despite perceived value, and (c) emerging practical solutions focused on feasible modular integration, interactive/case-based teaching, digital tools, and validated assessment approaches.

## DISCUSSION

Across the literature, EvMed is positioned as an integrative explanatory framework that complements proximate biomedical mechanisms by providing ‘ultimate’ explanations for vulnerability to disease (e.g. trade-offs, constraints, mismatch, host–pathogen coevolution) [1, 14]. This framing aligns with broader scholarship arguing that evolutionary biology can function as a basic medical science that connects otherwise disparate content and supports causal explanations in medicine [3, 31]. A notable development in the wider literature, which is highly relevant to curriculum design is the emphasis on structured explanatory frameworks that help learners avoid common misconceptions (e.g. genetic determinism, hyperadaptationism). One widely used example is Tinbergen’s four questions (mechanism, development, function, phylogeny), which have been proposed as a scaffold for integrating proximate and evolutionary explanations in medicine and [5, 7, 32]. Recent synthesis work in EvPsych similarly argues that Tinbergen’s framework clarifies explanatory targets and can guide clinically useful hypotheses [6]. It is also worth noting that structured interdisciplinary frameworks beyond Tinbergen's questions including those developed within the broader theory of interdisciplinarity may offer additional organizational resources for curriculum design, particularly where EvMed interfaces with behavioural science, psychology, and the social determinants of health. Exploring how such frameworks can be operationalized within medical education represents a direction for future conceptual and empirical work. Despite consistent conceptual support, the descriptive studies in this review converge on the conclusion that formal curricular integration remains limited or uneven [20–23]. This pattern is congruent with wider commentary that, although the medical relevance of evolutionary thinking is frequently acknowledged, implementation is constrained by curricular crowding, limited faculty expertise, and uncertainty about responsibility for teaching (e.g. medical school vs premedical education) [1, 31].

The North American survey literature is particularly explicit about an ‘importance–coverage’ discrepancy and about anticipated controversy acting as a brake on expansion [20, 21]. These observations align with education research showing that controversial perceived scientific topics often require careful instructional design and faculty development to support constructive engagement and conceptual change (e.g. addressing teleological reasoning and misunderstandings about natural selection) [33].

Both the included literature and external sources suggest that EvMed instruction often relies on high-yield clinical exemplars such as antibiotic resistance, cancer as somatic selection, and mismatch-related non-communicable diseases. These topics make evolutionary processes visibly relevant to clinical outcomes [1, 20]. The prominence of mismatch is also consistent with a substantial external literature linking modern disease burdens to discordance between evolved biology and contemporary environments [34].

The evaluated and implementation-focused studies in this review suggest that EvMed can be taught effectively using brief, interactive seminars, case-based modules, and blended/digital learning tools [26–29]. Importantly, these approaches appear designed to work *with* the constraints of crowded curricula rather than against them.

The broader medical education literature provides a useful lens for interpreting these strategies. Contemporary curricula increasingly emphasize competency-based medical education (CBME), outcomes, and assessment alignment [35]. In this context, the move within the EvMed literature toward clearly articulated learning objectives [15], consensus core principles [1, 6], and validated assessment tools [30] can be interpreted as an attempt to make EvMed compatible with CBME and similar quality assurance expectations.

The included literature suggests that acceptance and perceived relevance can vary by specialty and context. In women’s health, practising obstetrician–gynecologists expressed strong support for evolutionary biology as relevant to training [25]. In psychiatry, conceptual papers make a strong case that evolutionary biology is foundational for understanding emotion, behaviour, and psychopathology [9, 18]. However, the seminar evaluation by Baptista *et al*. [26] reported comparatively smaller gains and lower perceived relevance among psychiatrists than other clinician groups, suggesting that generic EvMed teaching may not automatically translate into psychiatric utility without careful alignment to formulation and clinical decision-making. Evolutionary psychiatrists support the idea that explicit use of explanatory frameworks (e.g. Tinbergen’s questions) may help translate evolutionary concepts into clinically actionable models in psychiatry [5, 6].

A consistent limitation across studies was most outcomes assessed are short-term (knowledge, attitudes, self-reported relevance), with limited evidence linking EvMed teaching to durable changes in longer term clinical thinking. This is not unique to EvMed but reflects a wider challenge in health professions education where downstream clinical impact is difficult to measure and often requires longitudinal, mixed-methods, and implementation-focused designs [35].

A further finding of this review is the relative scarcity of Type 3 evaluation studies. Although the conceptual and descriptive literature has matured substantially, only a small number of studies have formally evaluated educational interventions, and most of these relied on short-term outcomes such as learner satisfaction, self-rated knowledge, or immediate pre–post changes. This imbalance limits the field’s ability to make evidence-based claims about educational effectiveness and downstream clinical impact.

Future work should therefore prioritize rigorous evaluation designs, including longitudinal follow-up, comparison groups, and outcomes that capture higher-order clinical reasoning and application rather than attitudes alone. Without a stronger Type 3 evidence base, EvMed risks remaining conceptually persuasive but empirically under-justified within increasingly outcomes-driven and competency-based medical curricula.

### Strengths and limitations

#### Strengths

This review has several strengths. It employed a transparent and systematic scoping methodology, adhered to PRISMA-ScR guidance, and captured a broad range of education-focused literature across medicine. The scoping protocol was published on OSF [36]. Multiple reviewers were involved in the thematic synthesis which allowed integration of heterogeneous evidence while preserving distinctions between conceptual, descriptive, and evaluative contributions. Additionally consultations with experts in the field guided the review. This review brought together a multi-decade, multi-method literature, and mapped how the field has progressed from early curricular advocacy to consensus-based definitions of core content and the creation of evaluation tools. A further strength is the explicit categorization of papers into conceptual, descriptive, and implementation/evaluation types, which helps clarify how the evidence base functions and where the major gaps remain.

#### Limitations

This scoping review has several limitations. First, the search strategy focuses on studies that explicitly uses the terms ‘Evolutionary Medicine’ or ‘Evolutionary Psychiatry’, and it is therefore possible that educational initiatives incorporating evolutionary concepts without using this terminology will not be captured. Second, the review is limited to English-language publications, which may have exclude relevant studies published in other languages. Third, as is consistent with scoping review methodology, the review will not exclude studies based on methodological quality, and findings should therefore be interpreted as a mapping of the literature rather than an assessment of effectiveness. A related limitation is that the review captures only those educational activities that have been published or formally described in retrievable sources. It is very likely that EvMed and EvPsych are being taught at additional institutions worldwide without generating publications, reports, or web-accessible documentation. This means the review reflects the published evidence base rather than the full landscape of actual curricular activity, and the extent of real-world implementation may be meaningfully greater than the literature suggests. Finally, the heterogeneity of study designs and educational contexts will limited direct comparison across studies; however, this reflects the current state of the field and supports the need for further systematic evaluation. Additionally, consistent with scoping review methodology, no formal quality appraisal was undertaken, and the findings should not be interpreted as evidence of educational effectiveness.

This review also does not capture training in clinical or medical psychology, or in psychotherapy, which operate within distinct professional and educational structures and were outside the a priori scope of the review. Given that psychotherapy is integral to psychiatric treatment and that evolutionary perspectives are increasingly being applied to psychological formulation and therapy, this represents a meaningful gap in the broader literature. Future scoping or systematic reviews focused specifically on evolutionary approaches within psychological training pathways would be a valuable complement to the present work.

## CONCLUSION

This scoping review demonstrates that EvMed is supported by a coherent and increasingly standardized conceptual foundation, with broad agreement that evolutionary thinking offers integrative explanatory value for medical education. However, despite sustained advocacy and recognized relevance, curricular integration remains limited, uneven, and frequently peripheral, constrained by structural, cultural, and pedagogical barriers. The existing evaluation literature suggests that EvMed can be taught feasibly and acceptably using brief, clinically anchored, and modular approaches, but the current evidence base is insufficient to establish robust educational or clinical impact. Strengthening the field will require a shift from primarily conceptual and descriptive work toward more rigorous evaluation and implementation research. Without this progression, EvMed risks remaining an intellectually compelling but marginal component of medical education rather than a fully embedded explanatory science within modern medical curricula.

## Supplementary Material

EvMed_EvPsych_ScopingRv_Suppl_Info_11AUG26_eoag019

## Data Availability

The data underlying this article are derived from published sources cited within the article. No new primary data were generated or analysed as part of this study. Data extracted for the scoping review are available from the corresponding author upon reasonable request.
