# Supplementary material for: Evolutionary medicine and psychiatry in medical education: a scoping review
Source: Evol Med Public Health. 2026 Oct 5;14(1):1–13. doi: 10.1093/emph/eoag019 (PMC13644824; doi:10.1093/emph/eoag019)
Supplement: Supplementary file 1 [file evmed_evpsych_scopingrv_suppl_info_11aug26_eoag019.docx]

Evolutionary Medicine and Psychiatry in Medical Education: A Scoping Review

# S.1 – Detailed Search Strategy for Databases

**PubMed / MEDLINE**

1. "EvMed" OR "darwinian medicine" OR "EvPsych"
2. undergraduate OR "medical school" OR "college of medicine" OR "medical schools" OR psychiatry OR residency OR postgraduate
3. "medical education" OR curricula OR "educational programme" OR training OR teaching OR "clinical education"
4. #1 AND #2 AND #3
5. Filters applied: English language; publication date from 1990 onwards

**PsycINFO**

( "EvMed" OR "darwinian medicine"

OR "EvPsych" )

AND ( medical education OR curriculum OR teaching OR training )

AND ( psychiatry OR medical student* OR residency OR postgraduate )

**ERIC**

AB ( "EvMed" OR "darwinian medicine"

OR "EvPsych" )

AND TX ( undergraduate OR "medical school"

OR "college of medicine" OR "medical schools"

OR psychiatry OR residency )

AND TX ( "medical education" OR curricula

OR "educational programme" OR training OR teaching )

**SCOPUS**

( TITLE-ABS-KEY ( "EvMed" OR "darwinian medicine"

OR "EvPsych" )

AND TITLE-ABS-KEY ( undergraduate OR "medical school"

OR "college of medicine" OR "medical schools"

OR psychiatry OR residency OR postgraduate )

AND TITLE-ABS-KEY ( "medical education" OR curricula

OR "educational programme" OR training

OR teaching OR "clinical education" ) )

AND PUBYEAR > 1989
